# Unraveling the Transcriptomic Profiles of Large and Small Donkey Follicles

**DOI:** 10.3390/genes16050602

**Published:** 2025-05-20

**Authors:** Yanping Wang, Zihao Gao, Qiang Zhang, Xuchuan Guo, Wei Xia, Xinli Gu, Weibin Zeng

**Affiliations:** 1 College of Animal Science and Technology, Shihezi University, Shihezi 832003, China; wypdky@126.com (Y.W.); ripperzq@outlook.com (Q.Z.); bzxmgzzgxc@163.com (X.G.); zwbdky@126.com (W.Z.); 2 College of Animal Science and Technology, Hebei Agricultural University, Baoding 071001, China; 18730117292@163.com (Z.G.); xiaweihawaii@163.com (W.X.)

**Keywords:** Xinjiang donkey, granulosa cells, RNA-seq, *EMCN*, *SYT12*, *FOXO3*

## Abstract

Background: The diameter of mature follicles in donkeys is several times larger than in cattle and sheep, but the key genes responsible for maintaining follicular development and preventing apoptosis remain unclear. Methods: This study observed the process of donkey follicular development using ultrasound and analyzed the changes in common reproductive hormones in serum. Granulosa cells (GCs) were collected from large (mature follicles, diameter ≥ 37 mm) and small (atretic follicles, diameter 10–25 mm) follicles for sequencing to screen differentially expressed genes (DEGs) and signaling pathways influencing the development of mature follicles. The roles of selected genes were further validated in in vitro cultured GCs. Results: Donkey follicles exhibited rapid growth 5–7 days before ovulation, reaching maturity at a diameter of 37 mm. The maximum diameter of ovulatory follicles was approximately 40.7 mm, while non-ovulatory follicles began to undergo atresia when reaching about 25 mm. Serum reproductive hormone levels aligned with follicular developmental status. RNA sequencing identified 3291 DEGs between large and small follicles, with KEGG analysis highlighting enrichment in the PI3K-Akt signaling pathway, focal adhesion, amoebiasis, and cancer pathways. Lentiviral overexpression and interference assays targeting the DEGs *EMCN* and *SYT12* revealed that *EMCN* positively regulates *FOXO3*, a key gene in the PI3K-Akt pathway. Conclusions: The *EMCN* gene in mature donkey follicles regulates *FOXO3* in the PI3K-Akt signaling pathway, potentially inhibiting apoptosis in follicular granulosa cells and sustaining follicular development until ovulation. This study provides insights into the mechanisms underlying follicular development in donkeys.

## 1. Introduction

The donkey is one of the common traditional tools of labor [1]. However, due to the advancement of mechanization and urbanization, the role of donkeys in labor and transportation has been neglected, resulting in a significant decline in the domestic donkey population. Nevertheless, the skin and meat of donkeys possess commercial and medical value, making the captive breeding of donkeys a subject of considerable attention amongst researchers [2]. Despite this, donkey reproduction is hindered by low fertility rates and slow breeding returns [3]. Several factors influence donkey reproduction, including nutrition, follicular development, and control over ovulation timing. Unlike other mammals, donkey sex organs exhibit distinct characteristics—the cortex of donkey ovaries is located internally while the medulla is external. Notably, donkey follicles display insensitivity to the endogenous hormone FSH and exogenous hormone PMSG, and PMSG can solely augment the maximum diameter of the dominant follicle [4]. The diameter of ovulatory follicles varies significantly among different animal species: approximately 6 mm in ewes, 15 mm in cows, and reaching either 34.5 ± 1.3 mm [5] or 37.2 ± 0.83 mm [6] in donkeys. However, the mechanism by which mature donkey follicles develop to such a remarkably large size without undergoing apoptosis remains unclear.

Follicular waves, which are characterized by asynchronous development of small and large follicles, occur during the estrous cycle. Within this cycle, there is a process of dominant selection where certain follicles are chosen, while others degenerate [7]. Following dominant selection, small follicles develop into ovulatory follicles, whereas degenerated dominant follicles undergo regression. The surge of FSH has been found to be closely associated with the wave of folliculogenesis. As FSH levels rise, follicles increase in size [8]. The FSH surge typically occurs once or twice a week, during which the largest follicle in the small wave reaches its maximum diameter before regressing [4]. The synergy between FSH and E2 secreted by granulosa cells (GCs) is well established in promoting follicular development and ovulation. There has been increasing evidence supporting the positive role of GCs in supporting follicles. GCs serve as the fundamental functional unit of the oocyte, providing nutrients and signals for follicular development through gap junctions. They play a critical role in the entire process of follicular development, maturation, and ovulation [9]. It has been found that the overexpression of autophagy genes, such as *LC3-II*/*LC3-I*, in GCs contributes to the development of polycystic ovary syndrome (PCOS) [10]. The electroacupuncture-induced mechanism may involve the regulation of PI3K/Akt/Foxo3a expression in granulosa cells to improve the developmental microenvironment of oocytes and inhibit granulosa cell apoptosis, possibly contributing to an improved clinical pregnancy rate [11]. PI3K is a key member of the growth factor superfamily signaling pathway, and its downstream signaling molecule is Akt. Akt plays an important role in various biological processes, such as cell metabolism, cell cycle regulation, and cell growth and apoptosis [12].

RNA-Seq technology enables the analysis of expression level changes in all transcription products within a specific tissue or cell across various genetic variations, developmental stages, and treatment conditions. Additionally, it allows the identification of novel transcriptomes and variable splicing events, thereby providing a foundation for uncovering the molecular regulatory mechanisms underlying specific biological processes [13].

In this study, we utilized B-ultrasound to monitor follicular development in donkeys and applied RNA-Seq to sequence GCs collected from large (mature) and small (atretic) follicles of Xinjiang female donkeys. We then analyzed the differentially expressed genes (DEGs) enriched in functional pathways and identified *EMCN* and *SYT12* among the DEGs as potential regulators involved in maintaining follicular maturation through validation in granulosa cells.

## 2. Materials and Methods

### 2.1. Animals

In this experiment, six Xinjiang donkeys were selected from Jiu Chang Donkey Industry (Shihezi, China), aged 3 to 5 years old, weighing 130 to 150 kg, all of which were parturition donkeys. Before the start of the study, the reproductive tract was examined rectally by ultrasound and no reproductive tract disease was found, and all the females had ovulated normally at least once. All the test donkeys were numbered and were carefully tended throughout the trial period.

### 2.2. Measurement of Follicles and Hormones

Daily B-ultrasound monitoring was conducted to assess follicular growth, atresia, and ovulation in both ovaries. The diameter of the dominant follicle at the time of minimal rupture-induced ovulation served as the criterion to determine follicular maturation. Estrus in jennets was evaluated using the teaser method combined with external behavioral observations. Using a vacuum blood collection tube containing calcium ions as a coagulant, blood samples (10 mL) were collected daily from the jugular vein and centrifuged at 3500 rpm for 10 min, and the serum supernatant was collected. Concentrations of reproductive hormones (FSH, LH, E2, and P4) were measured using ELISA kits (Quanshi Jin Reagent Co., Ltd., Beijing, China), and their dynamic profiles were plotted.

### 2.3. Transcriptome Sequencing

#### 2.3.1. RNA Extraction

Based on follicular development, the largest follicle on the ovulatory ovary was classified as a large follicle (mature follicle, diameter ≥ 37 mm), while the largest regressing follicle (with arrested growth) on the non-ovulatory ovary was defined as a small follicle (atretic follicle, diameter 10–25 mm). Follicular fluid from large and small follicles of jennets was collected using an in vivo follicular fluid collection device (Honda Instruments Co., Ltd., Tokyo, Japan). Under a stereomicroscope (Olympus Corporation, Tokyo, Japan), cumulus–oocyte complex were removed, and the remaining fluid was transferred to a 15 mL centrifuge tube. After centrifugation at 1000 rpm for 10 min, the supernatant was discarded, and the pellet containing GCs was retained.

Total RNA was extracted using TRIzol reagent. RNA purification was performed according to the protocol of the Ultrapure RNA Kit (Kangwei Century Biotech Co., Ltd., Nanjing, China) for both large and small follicular GCs. RNA integrity was confirmed by 1% agarose gel electrophoresis, and samples with OD260/280 ratios between 1.8 and 2.0, as measured by a spectrophotometer (CWBiotech Co., Ltd., Beijing, China), were selected for sequencing.

#### 2.3.2. Library Preparation for Transcriptome Sequencing

After extracting the total RNA of the samples, the mRNA of donkey GCs was enriched by magnetic beads with Oligo (dT), and the mRNA was fragmented by adding an appropriate amount of interrupting reagent at a high temperature, and the interrupted mRNA was used as the template to synthesize one-stranded cDNA, and then two-stranded cDNA was synthesized by configuring a two-stranded synthesis system and was purified and recovered using the reverse transcription kit from Total Gold, with sticky end repair, the addition of base “A” to the 3′ end of cDNA, and the ligation of junctions, followed by fragment size selection and finally PCR amplification. The cDNA was purified and recovered using the Reverse Transcription Kit (TransGen Biotech Co., Ltd., Beijing, China), the sticky end was repaired, the base “A” was added to the 3′ end of the cDNA, and the junction was connected, then the fragment size was selected, and finally, PCR amplification was performed. The constructed library was analyzed by the Agilent 2100 Bioanalyzer (Agilent Technologies, Santa Clara, CA, USA) and ABI StepOnePlus Real-Time PCR System (Applied Biosystems, Waltham, MA, USA) for quality control and was sequenced after passing the test.

#### 2.3.3. Data Analysis and Quality Control

The raw data obtained from sequencing are called raw reads. First, the reads with low quality, splice contamination, and excessive unknown base N content are filtered out, and the filtered data are called clean reads. The Q20, Q30, and GC content (Q20 and Q30 are Phred scores, which stand for the quality of the sequencing, and GC indicates the G, C base in the sequenced percentage). All downstream analyses were based on clean, high-quality data. The clean reads were then compared to the reference genome, followed by de novo transcript prediction, SNP&INDEL (Applied Biosystems, Waltham, MA, USA), and differential splicing gene detection. After obtaining the new transcripts, the new transcripts with protein coding potential were added to the reference gene sequence to form a complete reference sequence, and then the gene expression levels were calculated.

#### 2.3.4. Gene Expression Analysis

Differential expression analyses were performed for three conditions or groups (3 biological replicates per condition) using the DESeq2 (v1.40.0, Bioconductor 3.19) in R 4.3.1. DESeq provides statistical routines for determining differential expression in numerical gene expression data using models based on the negative binomial distribution. The resulting *p*-values were adjusted using the method of Benjamini and Hochberg to control for false discovery rates. Genes with a DESeq-corrected *p*-value < 0.05 were differentially expressed genes. We screened for key genes associated with inhibiting apoptosis in mature follicular granulosa cells.

#### 2.3.5. GO and KEGG Enrichment Analysis of DEGs

Differentially expressed genes were analyzed for graphene oxide enrichment using the goseq R package (v1.54.0, Bioconductor 3.19) and corrected for gene length bias. GO terms with a corrected *p*-value (q-value) less than 0.05 were considered to be significantly enriched for differentially expressed genes. KEGG is a database resource for elucidating the higher-level functions and uses of biological systems (e.g., cells, organisms, and ecosystems) from molecular-level information, particularly large-scale molecular datasets generated by genome sequencing and other high-throughput experimental techniques. We used KOBAS (v3.0.3, installed via Bioconda) to detect the statistical enrichment of differentially expressed genes in the KEGG pathway (http://www.genome.jp/kegg/, accessed on 1 June 2024). We identified signaling pathways associated with the inhibition of apoptosis in mature follicular granulosa cells.

#### 2.3.6. RNA-Seq Validation and Functional Gene Screening

Ten differentially expressed genes (five upregulated and five downregulated) were selected from the DEG analysis results. The Primer Premier 5.0 software was used to design quantitative PCR primers based on the gene sequences of donkeys published at NCBI as templates. RT-qPCR was used to verify the RNA-seq results, and the primer synthesis and PCR product sequencing were performed by Shanghai Bioengineering. The results of RT-qPCR and RNA-seq were converted into log_2_ (Fold Change) to compare the expression trends of the two. Functional genes were selected for subsequent experiments based on gene function and transcriptome analysis of the sequencing data.

### 2.4. Intracellular Validation Assays

#### 2.4.1. Construction of *EMCN*, *SYT12* Overexpression, and Interference Lentiviral Vectors

The third generation donkey follicle GCs [14], cryopreserved in our laboratory, were thawed and cultured, and after spreading to 80~90%, trypsin digestion was performed to collect the cells, the total RNA of GCs was extracted by using Ultrapure RNA Kit (Kangwei Century Biotech Co., Ltd., Nanjing, China), the quality and concentration were detected by a spectrophotometer, and the RNA was reverse-transcribed to cDNA by the HiFiScript cDNA Synthesis Kit (Kangwei Century Biotech Co., Ltd., Nanjing, China).

Primers were designed according to the full-length coding sequences of donkey *EMCN* and *SYT12* genes published in the NCBI database, and the upstream and downstream primers were added to the homologous sequences on both sides of NotI and NsiI on the LV5 vector, respectively, which were used for the subcloning of the vector. After the completion of the PCR reaction, the target gene fragments were recovered by using agarose gel electrophoresis and cutting the gel. The LV5 vector was double-digested with NotI and NsiI and electrophoresed, and the DNA gel recovery kit was used to recover the linearized LV5 vector. The amplified fragments were recombinantly cloned into the linearized LV5 vector using ClonExpress^®^ Entry One Step Cloning Kit (Vazyme Biotech Co., Ltd., Nanjing, China), and then the recombinant overexpression plasmids were identified.

According to the principle of shRNA design, three RNA interference target sequences were designed with the LV3 vector using Designer 3.0 (GenePharma), respectively. “TTCAAGAGA” served as the stem–loop structure in the shRNA template to prevent the formation of termination signals. GATCC was added to the 5′ end of the sense strand template of LV3-shRNA to complement the sticky ends generated by BamHI digestion. AATTC was added to the 5′ end of the antisense strand template to complement the sticky ends formed by EcoRI digestion. The LV3 vector was linearized to construct interference vectors targeting the *EMCN* and *SYT12* genes, and the resulting plasmid was identified by single enzyme digestion with EcoRI. After completion, the recombinant plasmid, membrane protein expression plasmid (pVSV-G), and packaging plasmid (pGag/Pol, pRev) were added proportionally to package the viral vector. The virus concentrate was collected, dispensed into centrifuge tubes, and stored at −80 °C until required.

#### 2.4.2. Detection of Carrier Titer

Lentiviral plasmid DNA and packaging plasmid were infected with 293T cells, cultured for 72 h, and observed under a fluorescence inverted microscope; it was seen that GFP expression decreased with the increase in cell dilution. The viral titers of recombinant lentiviral overexpression vectors and shRNA vectors were detected by the doubling dilution method, and the viral titers of SYT12-*Equus asinus*, EMCN-*Equus asinus*, EMCN-ass-207, EMCN-ass-277, EMCN-ass-730, and SYT12-ass-742 were 1 × 10^8^ TU/mL, SYT12-ass-1310, SYT12-ass-875 were 2 × 10^8^ TU/mL, and LV5-NC, LV3-NC were 9 × 10^8^ TU/mL.

#### 2.4.3. RT-qPCR Determination of Relative mRNA Expression

Based on the NCBI database, β-actin (internal reference), *EMCN*, *SYT12*, *PTEN*, and *FOXO3* sequences were used. Specific primers were designed using Primer 5.0 and synthesized by Shanghai Bioengineering Company, and RT-qPCR was used to detect the expression level of target genes in donkey granulosa cells infected with lentivirus. The lentiviral vector group was used as the control group. The 2^−ΔΔCT^ method was used for the calculation of relative expression with the gene β-actin as the internal reference correction. ^ΔCt^ = Ct (target gene) − Ct (reference gene) and ^ΔΔCT^ = ^ΔCt^ (control group) − ^ΔCt^ (EA group).

### 2.5. Data Analytics

SPSS 21.0 software was used for statistical processing. All the experiments were repeated at least three times, and the experimental data were expressed as “mean ± standard deviation”. One-way analysis of variance (ANOVA) was used to compare the differences between the groups, and the differences between the data were deemed significant at *p* < 0.05 and *p* < 0.01.

## 3. Results

### 3.1. Follicle Development and Hormonal Changes

**Figure 1:**
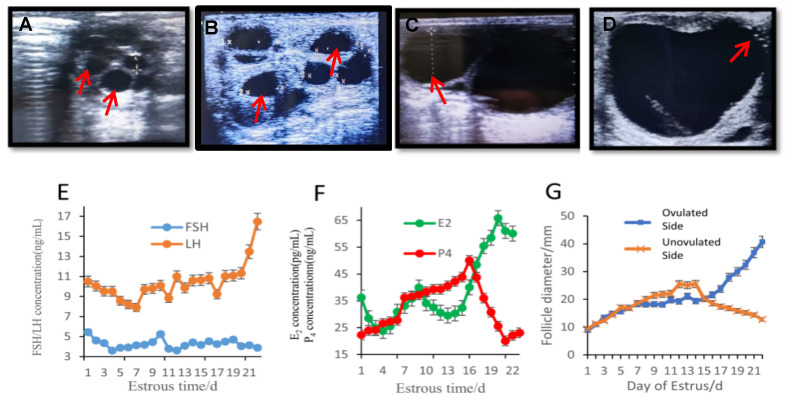
Measurement of follicles and reproductive hormones. (**A**) Small, dense follicles (The red arrow indicates) at the beginning of estrus. (**B**) Follicle growth squeezed to form irregular follicles (The red arrow indicates). (**C**) Subordinate follicles (The red arrow indicates) begin to undergo atresia and degeneration. (**D**) Dominant follicles protrude and extend (The red arrow indicates) in the ovulation nest direction. (**E**) Changes in FSH and LH during the estrous cycle of the Xinjiang donkey. (**F**) Changes in E2 and P4 during the estrous cycle of the Xinjiang donkey. (**G**) Diagram of maximum follicle development on the ovulatory side and on the anovulatory side of the ovary.

In Xinjiang female donkeys, the pre-estrus period is characterized by a high number of follicles, with round-shaped follicles being the most common (Figure 1A). As the follicles continue to grow, they start to exert pressure on each other, leading to changes in their shape. The follicles gradually transform into irregular shapes such as triangles (Figure 1B). During the middle and late stages of estrus, the follicles undergo further development and increase in size. Some subordinate follicles begin to undergo atresia and degeneration (Figure 1C). In the late estrus phase, the dominant follicle experiences rapid growth and occupies the entire ovary, and it begins to extend and protrude towards the ovulation site (Figure 1D,G).

These observations indicate that the presence of large follicles hinders the development of other follicles post-estrus, potentially resulting in the atresia and degeneration of smaller follicles. FSH reaches a peak on the 10th day of the estrus cycle, while LH levels reach their peak before ovulation (Figure 1E). The level of E2 rapidly increases with the growth of preovulatory follicles, while the concentration of P4 decreases before ovulation (Figure 1F). The levels of these common hormones are consistent with follicular development. On the 14th day of the estrus cycle, the largest follicle (approximately 27 mm in diameter) on the non-ovulatory side of the ovary stops growing and gradually begins to lock and regress. On the 16th day, the ovulatory follicle enters a rapid growth phase, growing at an average rate of 3.1 mm per day. At 21 days, its diameter is greater than 37 mm, and the follicle enters the mature stage. The follicle at the ovulation nest can rupture and ovulate. Some follicles that are far away from the ovulation site continue to grow and protrude towards the ovulation nest, with a diameter of about 40.7 mm before ovulation occurs (Figure 1D,G).

### 3.2. GC Gene Expression Differed Significantly Between Large and Small Follicles

**Figure 2:**
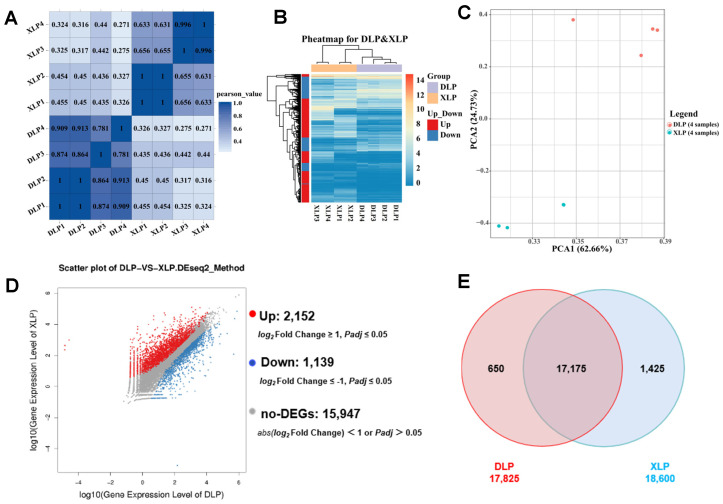
Gene association and expression analysis. (**A**) Correlation heatmap illustrating the correlation coefficients between samples. Blue shades indicate higher correlation. (**B**) DEG expression calorimetry displaying the log10-transformed expression levels of differential genes in different sample clusters. (**C**) Dimensionality reduction cluster analysis (PCA) plotting the disparity between samples after dimensionality reduction using principal components. The values in brackets represent the explained variance percentage. (**D**) Volcano plot of DEG, presenting log2-fold change on the *x*-axis and -log10 significance value on the *y*-axis. Red and blue dots correspond to upregulated and downregulated DEGs, respectively, while gray dots indicate non-DEGs. (**E**) Venn diagram showing the unique and shared genes expressed among samples and groups (note:DLP, large follicle; XLP, small follicle).

Initially, the samples of large- and small-follicle GCs were examined. The correlation coefficients between individual samples exceeded 0.3, indicating a significant correlation between the samples of large- and small-follicle GCs (Figure 2A). Based on the gene expression levels of individual samples, the DEGs between sample groups exhibited tight clustering, displaying low variability and good intra-group parallelism (Figure 2B). The volcano map shows that 3291 DEGs were screened, including 2152 upregulated genes and 1139 downregulated genes (Figure 2D). The GCs from small and large follicles exhibited substantial transcriptomic differences, indicating distinct cell types (Figure 2C). The GCs of small and large follicles contained 17,825 and 18,600 genes, respectively, with an overlap of 17,175 genes (Figure 2E). These findings suggest a high correlation between the genes of small and large follicles.

### 3.3. DEG Gene Ontology (GO) Functional Enrichment and KEGG Pathway Annotation

**Figure 3:**
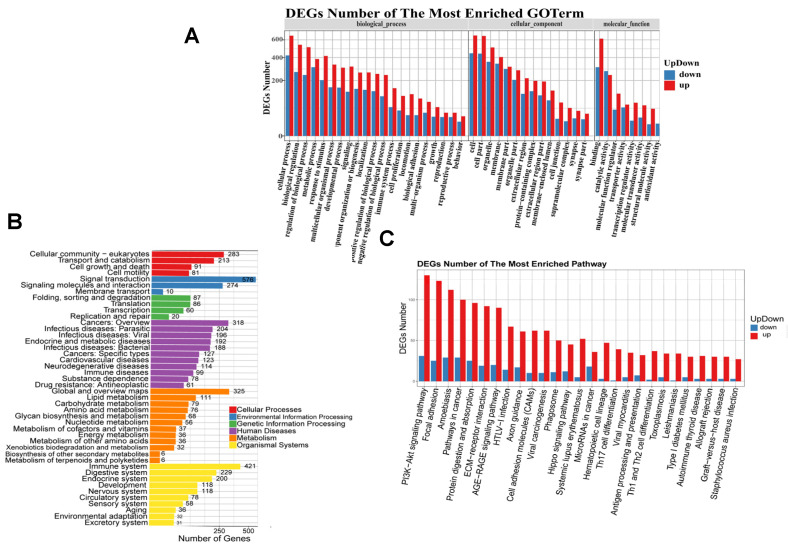

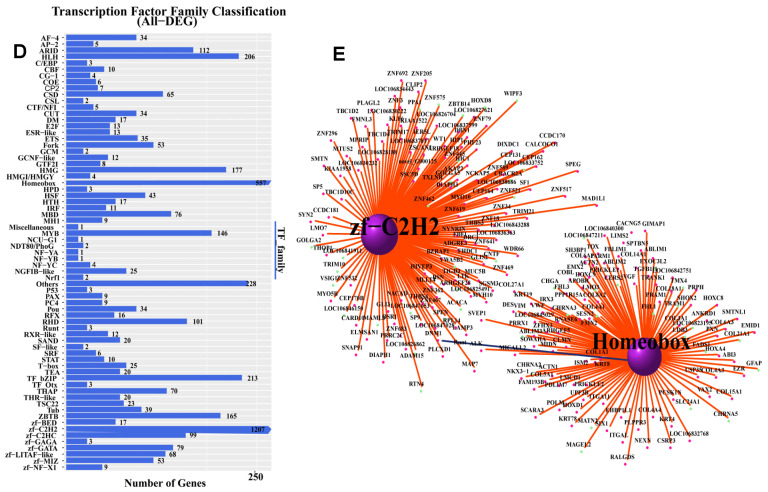
Differential gene function and channel analysis. (**A**) Differential gene GO function classification diagram. (**B**) Pathway classification of differential genes. (**C**) Differential gene regulation of enrichment pathways. (**D**) Classification of transcription factor families to which all genes belong. (**E**) TF-DEG network diagram.

The biological functions of the 3291 differentially expressed genes were analyzed through GO function classification and enrichment analysis. The results revealed that 1069 genes were assigned GO functional annotations, spanning 58 subcategories across three major categories: biological processes, cellular components, and molecular functions (Figure 3A). Within the 27 subcategories of biological processes, the DEGs showed significant enrichment in cellular processes, biological regulation, the regulation of biological processes, and metabolic processes. Within the 19 subcategories of cellular components, the differentially expressed genes (DEGs) exhibited significant enrichment in functional entries related to cells, cell parts, organelles, and membranes. Among the 12 subcategories of molecular function, there was a notable enrichment of functional entries including molecular binding, catalytic activity, molecular function regulation, and transporter activity. It can be observed that the GO functional enrichment results for cellular processes, cellular components, and molecular binding exhibit higher proportions among all the DEGs in that order.

Within living organisms, various genes collaborate with one another to carry out essential biological functions. In order to identify the key signaling pathways associated with DEGs, we performed classification and enrichment analysis on KEGG biological pathways. The analysis revealed that a total of 2145 genes were assigned KEGG pathway annotations (Figure 3B). It was observed that KEGG metabolic pathways associated with DEGs were classified into six branches: cellular processes, environmental information processing, genetic information processing, human diseases, metabolism, and organic systems.

The transcriptional capacity of DEGs encoding transcription factors (TFs) remains poorly understood. Thus, we conducted a prediction of DEGs with the potential to encode TFs. Our analysis revealed that the Homeobox and zf-c2h2 transcription factor families exhibited relatively high transcriptional capacity among the DEGs (Figure 3D). Subsequently, we constructed a network encompassing all TFs and DEGs, which indicated that the Homeobox and zf-c2h2 transcription factor families might serve as regulatory centers during follicular development (Figure 3E).

### 3.4. Screening of DEGs EMCN, and SYT12 and Construction of Viral Vectors

**Figure 4:**
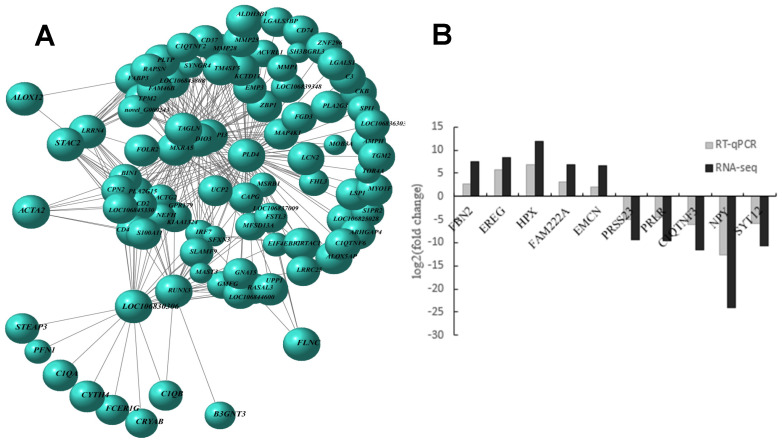
qPCR validation of selected differentially expressed mRNAs. (**A**) Gene co-expression network of WGCNA. (**B**) Validation of mRNA RNA-seq results by quantitative real-time PCR analysis. Expression levels of 9 mRNAs were quantified. Error bars are SEM, *n* = 4.

To further investigate the relationships between DEGs, we utilized the WGCNA (Weighted Correlation Network Analysis) software package to perform gene co-expression network analysis (Figure 4A). From donkey large and small follicles, we randomly selected five upregulated and five downregulated DEGs (*FBN2*, *EREG*, *HPX*, *FAM222A*, *EMCN*, *PRSS23*, *PRLR*, *C1QTNF3*, *NPY*, and *SYT12*) for validation using RT-qPCR, and their expression levels were compared with RNA-seq libraries (Figure 4A,B). We observed that the log2 (Fold Change) values of each gene aligned with the upregulation and downregulation patterns observed in the corresponding RNA-seq libraries. This convergence indicates the accuracy and reliability of the high-throughput sequencing results (Figure 4B). Among these genes, *FBN2* and *FAM222A* were significantly upregulated to a greater extent, while *HPX* and *EMCN* showed a relatively lower degree of upregulation. Likewise, *C1QTNF3* and *SYT12* displayed a higher degree of downregulation, whereas *PRLR* exhibited less pronounced downregulation. By combining the information from the GO and KEGG analysis of DEGs with the existing literature, we selected *EMCN* and *SYT12* genes for further functional validation, focusing on their interactions with key genes (*PTEN* and *FOXO3*) within the PI3K-Akt signaling pathway.

### 3.5. Lentiviral Vector Effectively Infects GCs

**Figure 5:**
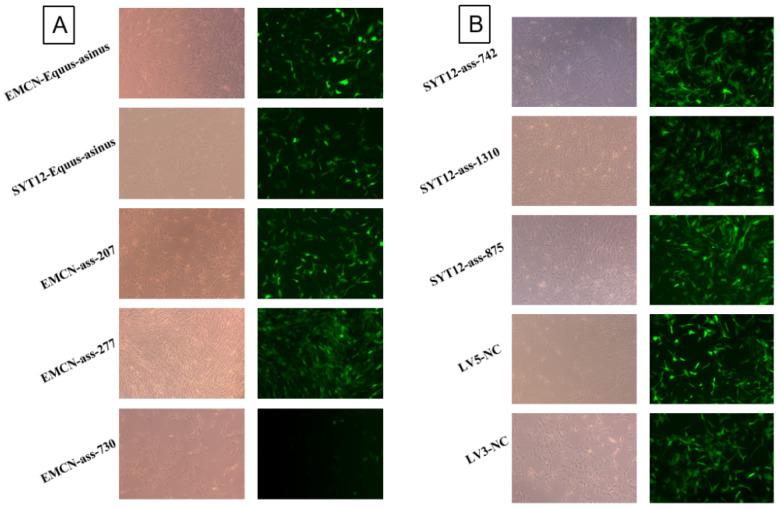
The functional validation of donkey follicular granulosa cells infected with recombinant lentivirus for 48 h. (**A**) Fluorescence expression of donkey follicular granulosa cells infected with *EMCN* recombinant lentivirus for 48 h (100×). (**B**) Fluorescence expression of donkey follicular granulosa cells infected with *SYT12* recombinant lentivirus for 48 h (100×).

In our experiment, lentiviruses were used to infect GCs of donkey follicles in each experimental group for 48 h. The fluorescence intensity was observed using a fluorescence microscope. In the overexpression group (SYT12-*Equus asinus*, EMCN-*Equus asinus*) and the negative control group (LV5-NC), green fluorescence was observed in the GCs (Figure 5A). In the interference group, with the exception of EMCN-ass-730, all the other groups (EMCN-ass-207, EMCN-ass-277, SYT12-ass-742, SYT12-ass-1310, and SYT12-ass-875), as well as the negative control group (LV3-NC), showed the expression of green fluorescent protein (Figure 5B). These results indicate that the lentiviral infection of donkey follicle GCs was successful.

### 3.6. EMCN Positively Modulates FOXO3

**Figure 6:**
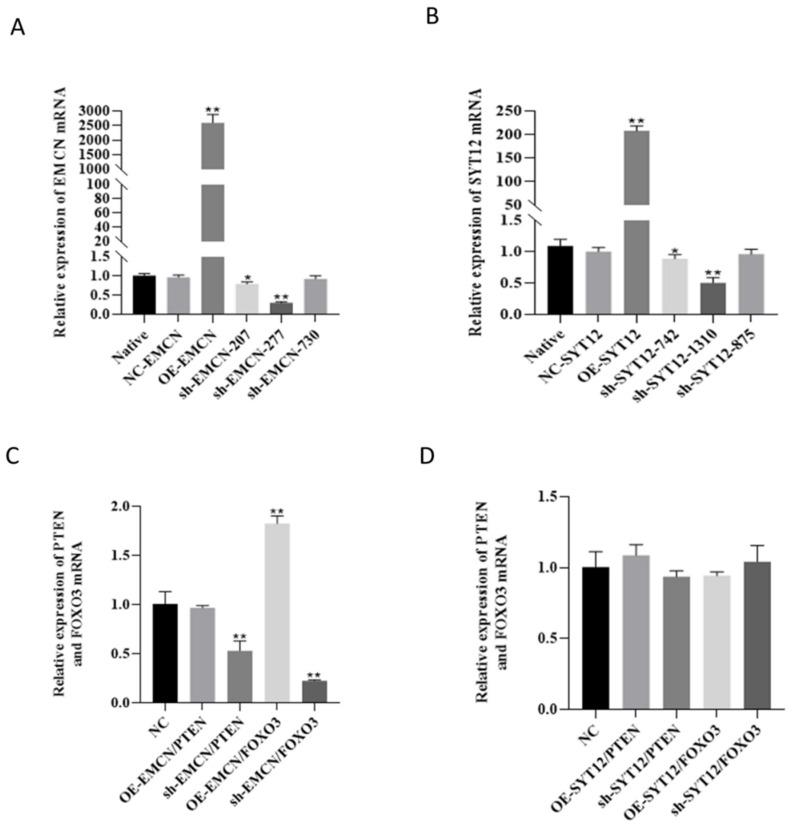
qPCR validation of selected differentially expressed mRNAs and functional validation of donkey follicular granulosa cells infected with recombinant lentivirus for 48 h. (**A**) *EMCN* expression results by quantitative real-time PCR analysis after OE or knockdown of *EMCN*. Error bars are SEM, n = 4. (**B**) *SYT12* expression results by quantitative real-time PCR analysis after OE or knockdown of SYT12. Error bars are SEM, n = 4. (**C**) *PTEN* and *FOXO3* expression of donkey follicular granulosa cells infected with *EMCN* OE/KD recombinant lentivirus for 48 h (100×). (**D**) *PTEN* and *FOXO3* expression of donkey follicular granulosa cells infected with *SYT12* OE/KD recombinant lentivirus for 48 h (100×). Note: “*” and “**” indicate significance (*p* < 0.05, *p* < 0.01).

We infected donkey follicular GCs with different titers of interfering lentiviral vectors and assessed the infection efficiency using RT-qPCR. In comparison to the control group (Native), the overexpression efficiency was found to be highest in the OE-EMCN group (*p* < 0.01). Additionally, the sh-EMCN-ass-277 knockdown efficiency was the most significant (*p* < 0.01) (Figure 6A). Among the overexpression groups, the OE-SYT12 group exhibited the highest mRNA overexpression efficiency (*p* < 0.01), whereas the sh-SYT12-ass-1310 knockdown efficiency was the most notable (*p* < 0.01) (Figure 6B). The statistical analysis indicated the significance of these differences in gene expression levels.

To investigate the role of *EMCN* and *SYT12* genes in the PI3K-Akt signaling pathway and their specific involvement in follicular development, we infected donkey follicle GCs with lentiviral vectors overexpressing the *EMCN* and *SYT12* genes, as well as interfering lentiviral vectors targeting these genes. The expression of key genes in the PI3K-Akt signaling pathway was detected using RT-qPCR. When the *EMCN* gene was overexpressed, there was no significant change in the expression of the PTEN gene. However, the expression of the *FOXO3* gene was significantly increased (*p* < 0.01). Conversely, when the *EMCN* gene was interfered with, the expression of both the *PTEN* and *FOXO3* genes was significantly reduced (*p* < 0.01), with a greater downregulation observed in *FOXO3* compared to *PTEN* (Figure 6C). In the case of the overexpression or interference of the *SYT12* gene, there were no significant changes observed in the expression of either the *PTEN* or *FOXO3* gene compared to the negative control group (Figure 6D).

## 4. Discussion

Throughout follicular development, various hormones cooperate or counteract each other to support follicular maturation and ovulation. In our study, the lowest level of E2 content was observed on day 15 of the estrous cycle in Xinjiang female donkeys, and an increase in P4 coincided with a decrease in E2. These findings demonstrate a resemblance to correlations found between P4 and E2 during the estrous cycle in Holstein cows, buffaloes, and Kazakh sheep [15]. Additionally, KanaiY confirmed the presence of this correlation in the trend in E2 content during the estrous cycle in buffaloes [15]. The plasma concentration of E2 exhibited a gradual increase from day 6 to day 2 prior to ovulation, while the concentration of luteinizing hormone rose from day 1 to day 3 post-ovulation [16]. These findings align with the outcomes of our current investigation. FSH exhibited a bimodal peak throughout the estrous period [7], whereas LH concentration gradually increased before ovulation and reached its peak on the first day after ovulation in female donkeys [17]. In a separate study by Gastal [18], focusing on both horses and donkeys, the LH peak was observed on the second day after ovulation, thus supporting our experimental findings. In this study, following the LH peaks, the follicles grew until they reached a maximum diameter of 40.7 mm before undergoing rupture and ovulation. Previous reports indicate that the dominant follicle in female donkeys grows at an average rate of around 2.7 mm per day following substantial follicular growth [19]. Our findings align with these reports. Our findings revealed that the pre-ovulation follicle diameter in female donkeys averaged approximately 40 mm, while in Martina Franca jennies and Texas black donkeys, it was 39.5 mm. In other donkey breeds, the pre-ovulation follicle diameter ranged from 36 to 41 mm [20], a variation likely influenced by factors such as donkey breed, age, and other factors.

It is intriguing that the largest follicle on the non-ovulatory side of the ovary stops growing at day 14 of the estrous cycle and begins to undergo atresia and degeneration. The development of the largest follicle on the ovulation side is not inhibited and continues to increase with the increase in LH concentration. When the follicle diameter reaches 37mm or more, it reaches the mature follicle stage, in which no significant inhibition of small follicular waves by dominant follicles can be observed [21]. This phenomenon may be attributed to reduced angiogenesis and the dependence of follicles on low concentrations of LH during the gonadotropin-dependent phase [22]. As sufficient LH stimulation occurs, the small follicular wave eventually develops into a main wave, supporting the sustained growth of multiple follicles [22]. Conversely, this contrasting situation may decrease the ability of large follicles to respond to hormones. However, Ginther suggests that changes in follicular morphology are a result of increased follicular size, pressure exerted by blocked ovaries limiting growth, and the squeezing effect caused by neighboring follicles or the corpus luteum [17]. Therefore, hormone levels may contribute to the asynchronous development of small and large follicles [23]. While we understand that hormones play a crucial role in influencing follicular development, the factors that regulate follicular activation and development still remain unclear.

Through the functional classification of differentially expressed genes in granulosa cells of large and small follicles in donkeys, it was found that these genes are mainly enriched in cellular processes, the regulation of biological processes, and metabolic processes. In the classification of cellular components, the enriched categories include cells, cellular compartments, organelles, and cellular membranes. In the classification of molecular functions, the main enriched categories are molecular binding, catalytic activity, molecular function regulation, and transporter activity. In gene expression analysis in mammalian ovarian tissues, the categories of cellular components, cell membranes, and molecular binding are highly represented [24]. Pathway enrichment analysis in DEGs can identify the main metabolic and signaling pathways that they are associated with, along with their interactions with other genes [25]. In this study, the differential genes *EMCN* and *SYT12* have a fold change of ≥2.00 and Padj ≤ 0.05, and 2145 genes annotated to KEGG are significantly enriched in four pathways: the PI3K-Akt signaling pathway, focal adhesion, amebiasis, and the cancer pathway, in this order. The PI3K/Akt pathway plays a crucial role in regulating cell proliferation, growth, and survival. During ovarian follicle development, this pathway is involved in maintaining the dormancy, survival, and activation of primordial follicles [25]. Furthermore, it promotes the proliferation of cells, and its local regulation is mediated by *FAK* [26]. *FAK* exhibits high expression in the ovary, particularly in the GCs of ovarian follicles, especially those in the antral stage [27]. Both the PI3K/Akt and focal adhesion signaling pathways participate in the regulation of primordial follicle activation and follicular development. However, further investigation of the specific regulatory mechanisms involving the genes within these pathways is warranted.

The PI3K/PTEN/Akt signaling pathway is crucial in controlling follicular quiescence and activation, playing a fundamental role in maintaining the dormant state of the primordial follicular reserve [28]. Within this pathway, the Forkhead transcription factor (FOXO3) is a key target regulated by PTEN/PI3K/AKT signaling [29]. Once activated, FOXO3 translocates from the nucleus to the cytoplasm, inhibiting its transcriptional function and leading to primordial follicle activation [30,31]. FOXO3 acts as a guardian of the primordial follicular pool, enhancing the ovarian reserve and preserving fertility [32]. Mouse models deficient in *FOXO3* show the premature activation of primordial follicles during the neonatal period, resulting in follicle loss and premature ovarian failure [33]. Elevated PI3K/AKT activity leads to the hyperactivation of primordial follicles, causing a significant reduction in follicle number and accelerating ovarian aging. The overphosphorylation of FOXO3 following PTEN inhibition prevents follicle growth [34]. In primordial follicles, Akt and mTOR phosphorylation initiates the activation of dormant follicles, while PTEN induction and FOXO3 activation (nuclear expression) help maintain follicular dormancy [35]. *EMCN*, a regulatory gene upstream of *FOXO3*, has been found to play a significant role in these processes. The overexpression of EMCN leads to increased FOXO3 expression. EMCN promotes the internalization of ligand-bound VEGFR2, which is involved in maintaining the functional state of the follicle through a healthy vascular supply [36]. Vascular permeability, mediated by VEGF, is particularly important in large follicles [37]. Thus, the regulatory roles of EMCN and FOXO3 are critical in follicular development and ovulation.

Notably, *SYT12* activates the PI3K/AKT/mTOR signaling pathway by phosphorylating *PIK3R3*. Although the SYT family primarily function as calcium ion receptors, SYT12 also plays a role in cancer cell proliferation, metastasis, and apoptosis [38,39]. However, no significant changes were observed in the expression of *PTEN* and *FOXO3* upon the overexpression or interference of *SYT12*. This suggests that *SYT12* may not be a key gene target of GCs in this context. In summary, follicular development in Xinjiang female donkeys appears to be positively regulated by the effect of *EMCN* on *FOXO3* through GCs. This regulation influences hormonal stimulation by modulating vascular permeability. On the other hand, *SYT12* does not seem to have a significant effect on this process.

## 5. Conclusions

Large follicles on the ovulatory side reach 37 mm, they enter the mature stage, and the maximum diameter of ovulatory follicles is about 40.7 mm. The *EMCN* gene in mature donkey follicles regulates the *FOXO3* gene in the PI3K Akt signaling pathway, inhibiting the apoptosis of granulosa cells and maintaining the growth and development of mature follicles until they rupture and ovulate. This result provides a basis for the specific mechanism of follicular growth and development in female donkeys.

## Data Availability

Data presented in this study are available upon request from the corresponding author.
